# The motivation against change in male methamphetamine users in the compulsory detoxification setting

**DOI:** 10.3389/fpsyt.2023.1022926

**Published:** 2023-02-06

**Authors:** Wenwen Shen, Longhui Li, Yue Liu, Xiaohu Xie, Weisheng Chen, Huifen Liu, Wenwu Zhang, Yu Liu, Haihang Yu, Wenhua Zhou

**Affiliations:** ^1^Laboratory of Behavioral Neuroscience, Ningbo Kangning Hospital, School of Medicine, Ningbo University, Ningbo, Zhejiang, China; ^2^Key Laboratory of Addiction Research of Zhejiang Province, Ningbo, Zhejiang, China; ^3^Ningbo Clinical Research Center for Psychiatry and Psychological Disorders, Ningbo, Zhejiang, China; ^4^School of Medicine, Ningbo University, Ningbo, Zhejiang, China; ^5^Ningbo Kangning Hospital, School of Medicine, Ningbo University, Ningbo, Zhejiang, China

**Keywords:** motivation against change, addiction, methamphetamine, compulsory detoxification, craving

## Abstract

**Aims:**

The study was designed to develop a measurement for the motivation for and against change in methamphetamine users in the compulsory detoxification setting.

**Design:**

This is a cross-sectional study.

**Setting:**

The study was carried out in a compulsory detoxification center for male drug users in China.

**Participants:**

A total of 228 male methamphetamine users who had undergone the program for at least 30 days.

**Measurements:**

The motivation for/against change relating to compulsory detoxification was carried out using the Likert scale. A series of questionnaires were filled out by the participants, including the Egna Minnen Beträffande Uppfostran for rearing style, the Barratt Impulsiveness Scale-11, the adult ADHD self-report scale, and the Pittsburgh sleep quality index. Participants were also asked to recall the withdrawal symptoms before the program and to rate their current craving levels.

**Findings:**

Motivations were grouped into three factors, namely, the expectation to use drugs upon the completion of the program (factor 1), the disagreement with the compulsory setting (factor 2), and the motivation to quit drug use (factor 3). Cronbach's alpha values were 0.8037, 0.8049, and 0.6292, respectively. The structural equation model showed that the overall motivation was characterized by motivation against change rather than that for change. The overall motivation was also directly affected by the current craving level and indirectly affected by the severity of addiction, paternal authoritarian upbringing style, and ADHD traits.

**Conclusion:**

This study provided a measurement of motivation for and against change in subjects with drug misconduct and suggested that the motivation against change may disclose more psychological barriers than the motivation for change.

## 1. Introduction

Prolonged methamphetamine use not only resulted in an imbalance of the reward system and the vulnerability to psychosis but also resulted in a decline in cognitive and social functions. In China, where methamphetamine has been the dominant drug of abuse in recent decades, various medical measures and psychosocial interventions have been introduced with close examination, such as transcranial magnetic stimulation (1), transcranial direct current stimulation (2), and substance use monitoring using hair samples (3), mainly under a compulsory treatment setting.

Compulsory detoxification (CD) is an important component of the current drug control system in China. It has been offered to individuals who have been found using illicit drugs repeatedly. CD usually lasted for 1–3 years, with an annual assessment of the physical and psychological health condition. It creates a drug-free environment and guarantees enough time for individuals to overcome drug-related distress during the withdrawal and protracted period, so that the impulse to use drugs could be greatly reduced. Indeed, the self-reported basal craving level was stably low in subjects with more than 3-month detoxification (4). Yet, cue-related cravings could be evoked even after long-term abstinence (5). The self-reported anticipation of relapse was high in a drug-isolated setting (6) but has improved in recent years (7).

Motivation for change is considered a pivotal prerequisite in successful recovery from addiction. It could predict future addiction severity, as shown in a study of alcoholism (8). Motivational interviewing helps patients resolve their ambivalence and establish personalized motivation for change (9). The studies of motivation for change are still limited in the CD settings. In an early study, Zhu et al. (6) reported that only six out of 360 subjects explicitly expressed the willingness to quit heroin use permanently, and almost all acknowledged the “inevitable” relapse. This study was carried out around the year when the regulation of drug use decriminalization (10) was carried out in China. In the most recent study, the researchers showed that the average motivation for drug rehabilitation could be rated as a medium using the Stages of Change Readiness and Treatment Eagerness Scale (SOCRATES) (7); yet, the statistics were slightly lower than a survey carried out in the similar setting in 2007 (11). Therefore, there seemed to be a discrepancy between reports of low motivation by Zhu et al. (6) and those of medium motivation using SOCRATES (7, 11). Moreover, the SOCRATES seemed not enough to predict relapse in post-CD individuals regardless of social worker services (7), suggesting a loss of validity for the questionnaire related to CD settings.

The most important difference between these studies was that the former was done in a semi-structural free-talk style, while the latter two were carried out by questionnaire filling. By examining the content of SOCRATES, we believed that one important component of motivation was overlooked in the questionnaire: the motivation against change. The dimensions in SOCRATES were constructed under the consensus that substance use is harmful and should be stopped. It may, therefore, create an implicit social pressure and, therefore, the social desirability bias, especially when illicit drug use is believed as immoral by the public. This may particularly be true for subjects under the CD settings, in which they had a deep worry about the disclosure of their own thoughts. A similar concern has been raised by Lombardi et al. (12), who measured individual statements against change for patients with generalized anxiety and found that participants responding to cognitive–behavioral therapy differed in counter-change talk but not in change talk. Counter-change talk has also been found to be more predictive than change talk in reducing hazardous drinking (13) and substance use (14).

In the Motivational Interviewing Skills Code (MISC) (15), change talk and counter-change talk was grouped into three categories, namely, (1) commitment or explicit statement of motivation for/against change; (2) rationales for/against change; and (3) willingness or inclination to/against change. The content of counter-change talk would be featured by the shared environment setting and conditions. For example, for veterans with drinking issues, the motivation against change mainly included the benefits of alcohol use, especially in coping with post-traumatic stress disorder. For individuals in the CD setting, the motivation against change would most likely be any kind of commitment and rationale for drug use. Therefore, we designed a questionnaire to include both change and counter-change statements for CD individuals, trying the capture the whole picture of motivation for/against change.

The motivation for/against change could be associated with a psychosocial background and self-images. For example, in patients with eating disorders, motivation for change could be higher in patients with less body dissatisfaction, more adaptive parent–adolescent relationships, and fewer depressive symptoms (16). Individuals with attention-deficit/hyperactivity disorder (ADHD) seemed more likely to develop various addictive behaviors (17, 18). Poorer upbringing in the environment, including childhood trauma and neglect, may lead to severe cases of addictive disorders (19, 20). Understanding and untangling these influence factors could help build the relationship between the service providers and the drug users. To explore possible influence factors and their relationship with drug-related motivations, we investigated the inattentive and impulsive traits, upbringing environments, and features of methamphetamine use, in addition to the survey of motivation for/against change. The survey for motivation was carried out in a self-designed questionnaire that contained both change talk and counter-change talk that relates to the specific CD settings.

**Figure 1:**
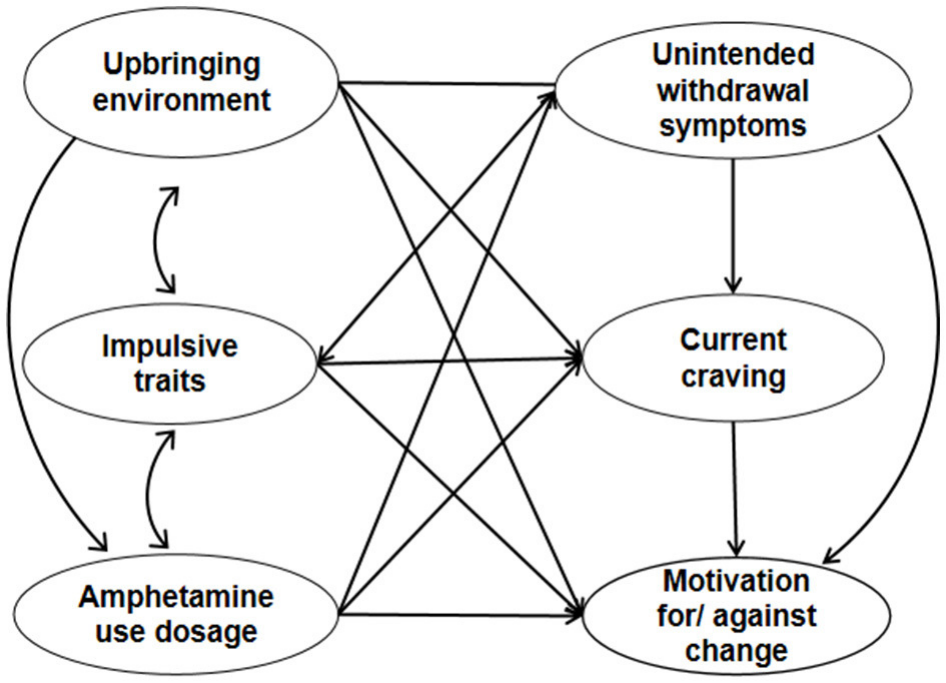
Conceptual model of the relationship of motivation with the upbringing environment, impulsivity traits, and drug intoxication.

The assumptions of the study were as follows: (1) Motivation against change could better explain the psychosocial barriers of the patients. (2) Drug-related features, personal traits, and psychosocial background, such as upbringing experiences, contribute to the formation of motivation. (3) Drug use experience, personal traits, and upbringing environment had mutual interactions toward each other. The conceptual model is shown in Figure 1.

## 2. Materials and methods

Participants were recruited from the Zhangjiang Compulsory Detoxification Center for men in Zhangjiang Province in China in July 2013. The study was part of a program carried out to investigate the effectiveness of the local CD program, which was approved by the Ethics Committee of Ningbo Addiction Research and Treatment Center. The inclusion criteria are (1) age>18 years, (2) able to express their willingness to participate, and (3) methamphetamine as the main drug of abuse. The exclusion criteria are (1) unwilling to take part in the study or unwilling to sign the informed consent and (2) having unstable conditions such as major depression or acute psychosis. The voluntary nature of the study and anonymity were emphasized throughout the study, and subjects were informed that their responses related to the study would not be used as an evaluation of their performance. People who were unwilling to participate were allowed to stay in the investigation room until they felt safe to leave.

After giving written informed consent, 228 male methamphetamine users accepted a structural interview carried out by the researchers face to face, which focused on (1) demographic and socioeconomic information, (2) detailed features of drug use, (3) experiences of childhood trauma, (4) subjective feelings of program settings, and so on. Several questionnaires were self-rated by the participants.

### 2.1. Questionnaires

#### 2.1.1. Egna Minnen Beträffande Uppfostran (EMBU) for rearing style

The EMBU is an 81-item self-report inventory for individuals to rate their experience with the major caretakers (21). The Chinese version of the EMBU (EMBU-CV) was revised by Yue et al. (22). It has 58 items with a 4-point Likert rating. It consists of six factors of paternal parenting style (emotional warmth, punitive, favoring subjects, rejection, control, and over-protection) and five factors of maternal parenting style (emotional warmth, punitive, favoring subjects, rejection, and control/over-protection). Cronbach's alphas of the EMBU-CV ranged from 0.46 to 0.85, and the test–retest reliability coefficients ranged from 0.58 to 0.82. In this study, the sub-scale of favoring subjects was not included because there was a large proportion of “single child” in the sample.

#### 2.1.2. Barratt impulsiveness scale-11 (Chinese version)

The Chinese version was revised by Li et al. (23) with 30 items. It is a 4-point Likert scale. Cronbach's alphas were 0.77–0.89, and the test–retest reliability coefficients were 0.68–0.89. It has three factors, namely, non-planning, motor impulsivity, and attention.

#### 2.1.3. Adult ADHD self-report scale (ASRS)

The ASRS is an 18-item questionnaire developed by Kessler et al. (24). It is a five-level Likert scale assessing the frequency of occurrence of each of the symptoms. It grouped the symptoms into the hyperactivity cohort and impulsivity cohort according to the DSM-IV criterion A symptoms of adult ADHD. The sensitivity of the scale was 56.3%, and the specificity was 98.3%.

#### 2.1.4. Rating of symptoms during unintended withdrawal and current craving levels

Participants were asked to recall and evaluate their symptoms in the last year of methamphetamine use. Mostly, individuals would experience some kind of withdrawal symptoms if they were out of the drug, during which time they had no intention to quit drug use. These symptoms were defined as unintended withdrawal symptoms in this study. The levels of fatigue, craving, and depression during unintended withdrawal were investigated by a 10-centimeter visual analog scale (VAS), with 0 representing not existent and 10 representing extremely strong discomfort. The levels of insomnia, hypersomnia, binge eating, psychomotor hyperactivity, and psychomotor retardation were evaluated using the Likert scale, where 1 represents not existent, 2 as mild and not affecting life quality, 3 as feeling bothersome, and 4 as extremely bothered. The current craving level was assessed using another 10-cm VAS. Participants were instructed to rate their current longing for methamphetamine without any deliberate imaging of methamphetamine using the setting.

#### 2.1.5. Pittsburgh sleep quality index (PSQI)

The Pittsburgh sleep quality index is a questionnaire with 18 self-rated items (25). It has been the most used questionnaire for subjective sleep quality. The Chinese version of PSQI was developed in 1996 (26), and a global cut-off value was set to 7 to distinguish normal subjects from patients with sleep quality problems in this version.

#### 2.1.6. Symptom checklist 90 (SCL-90)

The symptom checklist 90 is a psychosomatic screening scale that has been widely used in China since 1984 (27). It is a 5-degree Likert scale assessing the severity of certain psychiatric and somatic symptoms. In this study, denial of a symptom would be rated as 0, and an extremely severe symptom would be rated as 4. Only the averaged total score was included in the correlational analysis.

#### 2.1.7. The motivation for/against change relating to compulsory detoxification (MFACD)

The motivation for/against change relating to compulsory detoxification was designed by the researchers to investigate the motivation for and against continuous drug quitting after the CD program. The MFACD has 15 items and uses a 4-degree Likert scale, from 1 as strongly disagree to 4 as strongly agree. Example items include “I feel compulsory program is a violation to my personal rights,” “I will use drug once I finish the compulsory detoxification,” “I know people who have health issues concerning drug use, and I am afraid that the same would happen to me.”

### 2.2. Statistical analysis

The data were collected in Microsoft Excel forms and processed with Python. The first step was to describe the range, distribution, mean, median, and missing values of each item. Then the missing values were filled in with neighboring values. MFADU was analyzed with exploratory factor analysis using the FactorAnalyzer package. Kaiser–Meyer–Olkin's measure of sampling adequacy and Bartlett's test were run to measure the construct validity of the questionnaire, and the extracted factors with an eigenvalue above 1.0 were examined. Items with a factor loading above 0.4 were included in the relative factor if their loading were lower than 0.3 in any other factors. Items with more than one loading above 0.4 were included only in the factor with higher loading. Items with all factors loading below 0.3 were not included in any of the factors. Structural equation analysis was carried out with a semopy package (28). To abstract the factors consistent with theoretical models, principal component analysis was run to extract unintended withdrawal symptoms, ADHD and impulsivity traits, and authoritarian type of paternal rearing style. Then, the whole model was constructed and fitted, with necessary adjustments and evaluation. Model fitting was carried out by Sequential Least Squares Programming (SLSQP). Correlational data were plotted using matplotlib and seaborn packages.

## 3. Results

The main information of the 228 participants is listed in Table 1. They aged between 20 and 56 years and were kept in a male detoxification center for at least 30 days (mean 303 ± 173 days, ranging from 30 to 879 days). The most common education level of these patients was middle school, which resembles the education level in the national population sample survey (29). The majority of people had no stable relationship or stable occupation. Patients had been using methamphetamine for a median of 2.5 years, with a median dosage of 130 g in their last year before the program. Among them, 98 patients reported experiencing psychotic symptoms during use. Almost all participants met the diagnostic criteria of methamphetamine dependence or abuse of the DSM-IV, yet 11 patients met only the criteria of recreational methamphetamine use according to their own narrative.

**Table 1:** General information on the 228 male methamphetamine users in the CD program.

**Items**	**Description [M + SD, or median (min, max)]**
Age	33.15 ± 7.27, median 31.2 (20.6, 55.7)
Duration of the program (days)	303 ± 173
Body mass index (kg/m^2^)	23.4 ± 2.9
Education (years)	9 (0, 16)
Marital status: single (%)	72.8%
Occupation	No job: 97 Self-employed: 82 Peasant: 20 Employed: 19 Serve in recreational places: 10
**Drug use features**	
Age of drug use onset (years)	27.8 ± 7.5
Years of MA use	2.9 ± 2.3, 2.5 (0.1, 16.7)
Amount of MA dose in the last year of using (g)	197.5 ± 196.3, 130 (0, 1092.0)
History of psychosis symptoms	Delusion: 73 Hallucination: 78 Either delusion or hallucination: 98
**Barratt impulsivity scale 11**	
Cognitive impulsivity	4.87 ± 1.42, 4.75 (1.5, 10)
Non-planning	5.37 ± 1.78, 5.25 (0, 10)
Motor Impulsivity	3.98 ± 1.62, 3.88 (0, 8.25)
**ASRS**	
Inattentive	3.69 ± 1.26, 3.61(0, 7.5)
Overactivity	3.39 ± 1.35, 3.33 (0, 7.5)
**EMBU: Paternal**	
Emotional warmth	5.11 ± 1.95, 5.26(0, 9.65)
Punitive	2.11 ± 1.92, 1.38 (0, 9.44)
Deny	2.33 ± 1.67, 2.22 (0, 10.0)
Control	3.59 ± 1.45, 3.67(0, 8.33)
Over-protection	3.46 ± 1.67, 3.33(0, 8.89)
**EMBU: Maternal**	
Emotional warmth	5.58 ± 1.90, 5.53(0.70, 9.48)
Punitive	1.38 ± 1.73, 0.74 (0, 9.63)
Deny	2.08 ± 1.69, 1.67(0, 7.5)
Control and over-protection	4.13 ± 1.57, 3.96(0, 9.17)
PSQI	5.59 + 3.16, 5.0(0–15.5)
SCL-90 total score	1.60 ± 1.25, 1.33(0, 6.03)
Current craving	1.71 + 2.56, 0.3(0–10.0)
**MFACD**	
Factor 1: The expectation to use drug upon the completion of the program (drug lust)	5.86 ± 2.56
Factor 2: The disagreement with the compulsory setting	3.10 ± 2.39
Factor 3: The motivation to quit drug use	7.76 ± 2.04

Most statistics in the questionnaires were transformed into standard scores, of which 10 was the allowed highest mark in the sub-scale, and 0 was the allowed lowest mark.

Methamphetamine users showed relatively high scores in BIS-11, while their ASRS scores were all below the line of suspected ADHD. PSQI scores suggested that 25.4% of participants had sleeping problems.

**Figure 2:**
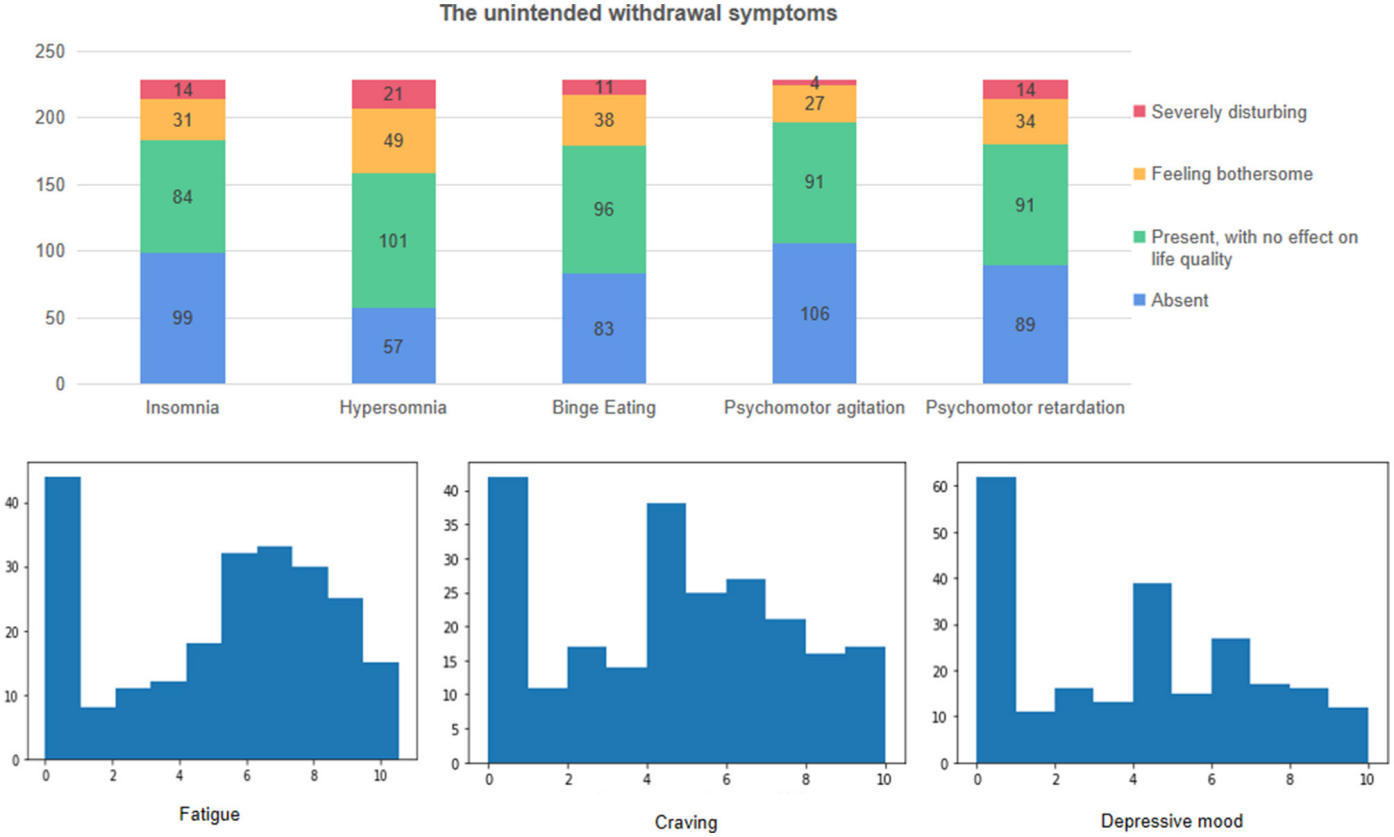
Unintended withdrawal symptoms were reported by the participants.

The unintended withdrawal symptoms (Figure 2) were acknowledged by most of the participants. The most reflected symptoms were fatigue and craving. The craving level at the time of investigation was much lower, with a median score of 0.32 out of 10.

The motivation for/against change relating to compulsory detoxification was clustered into three factors. Factor 1 was the expectation of using the drug after they finish the program; factor 2 was the disagreement with the CD program; and factor 3 was the motivation to quit drug use. Cronbach's alpha values were 0.8037, 0.8049, and 0.6292, respectively. Basically, both factors 1 and 2 represented the motivation against change, while factor 3 represented the motivation for change.

**Figure 3:**
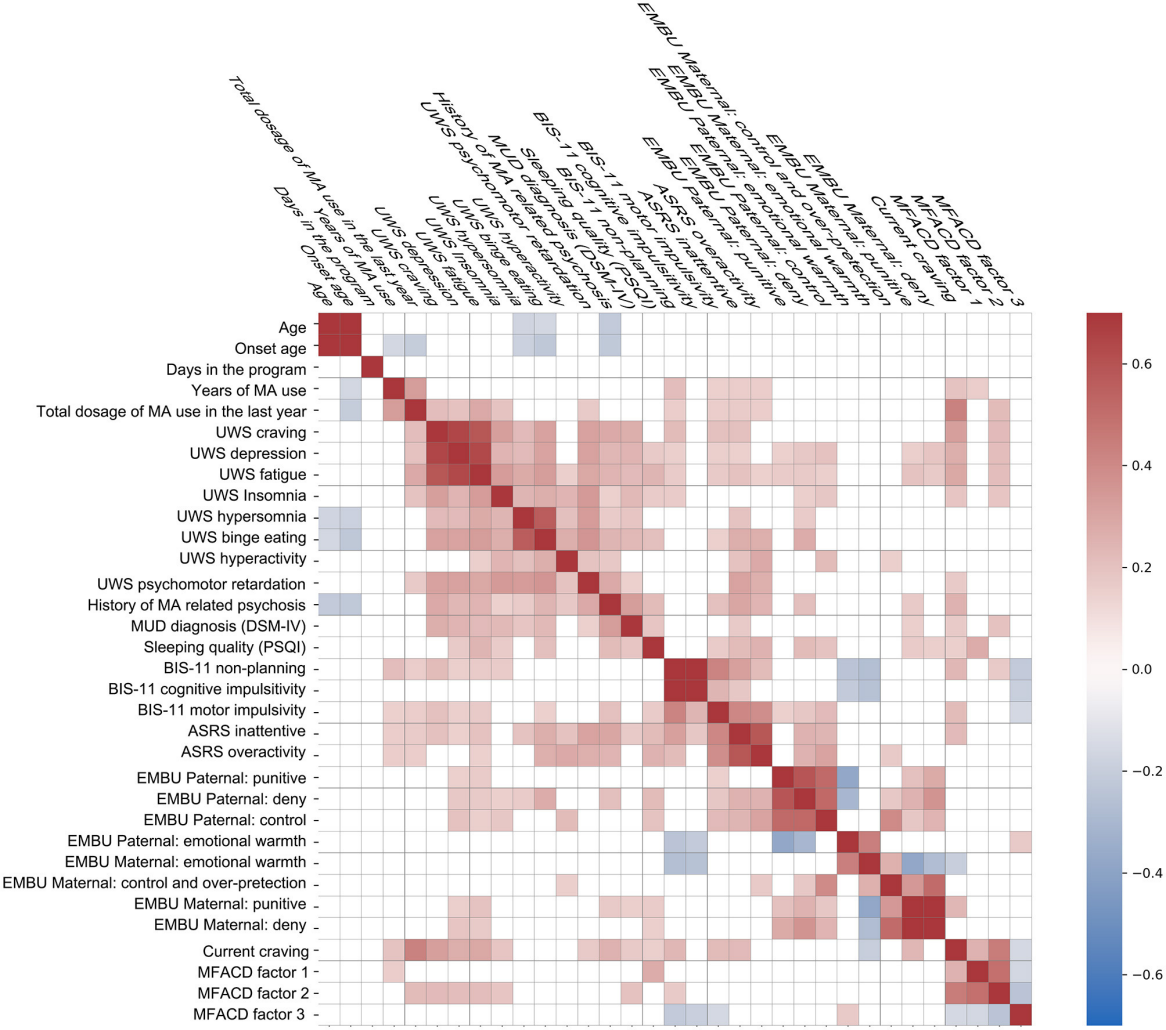
Correlational map of the motivations, upbringing environment, impulsivity traits, and drug intoxication. UWS: unintended withdrawal symptoms. MUD: methamphetamine use disorders. BIS-11, Barratt Impulsiveness Scale = 11. MFACD, the motivation for/against change relating to compulsory detoxification. Pearson correlation coefficients >0.15 (or <-0.15) were colored on the map.

The correlational analysis is shown in Figure 3 and Supplementary Table 1. Drug use features, psychological measures, and psychiatric symptoms were closely related to each other. The duration of the program was not related to any psychological or psychiatric indices we investigated. In brief, MFACD factor 2 was correlated with the total MA using years, PSQI scores, craving, BIS-11 scores, and EMBU parental warmth. Parental authoritarian rearing style (deny, control, and punitive) was also closely related to the unintended withdrawal symptoms, BIS-11, and ASRS scores.

**Figure 4:**
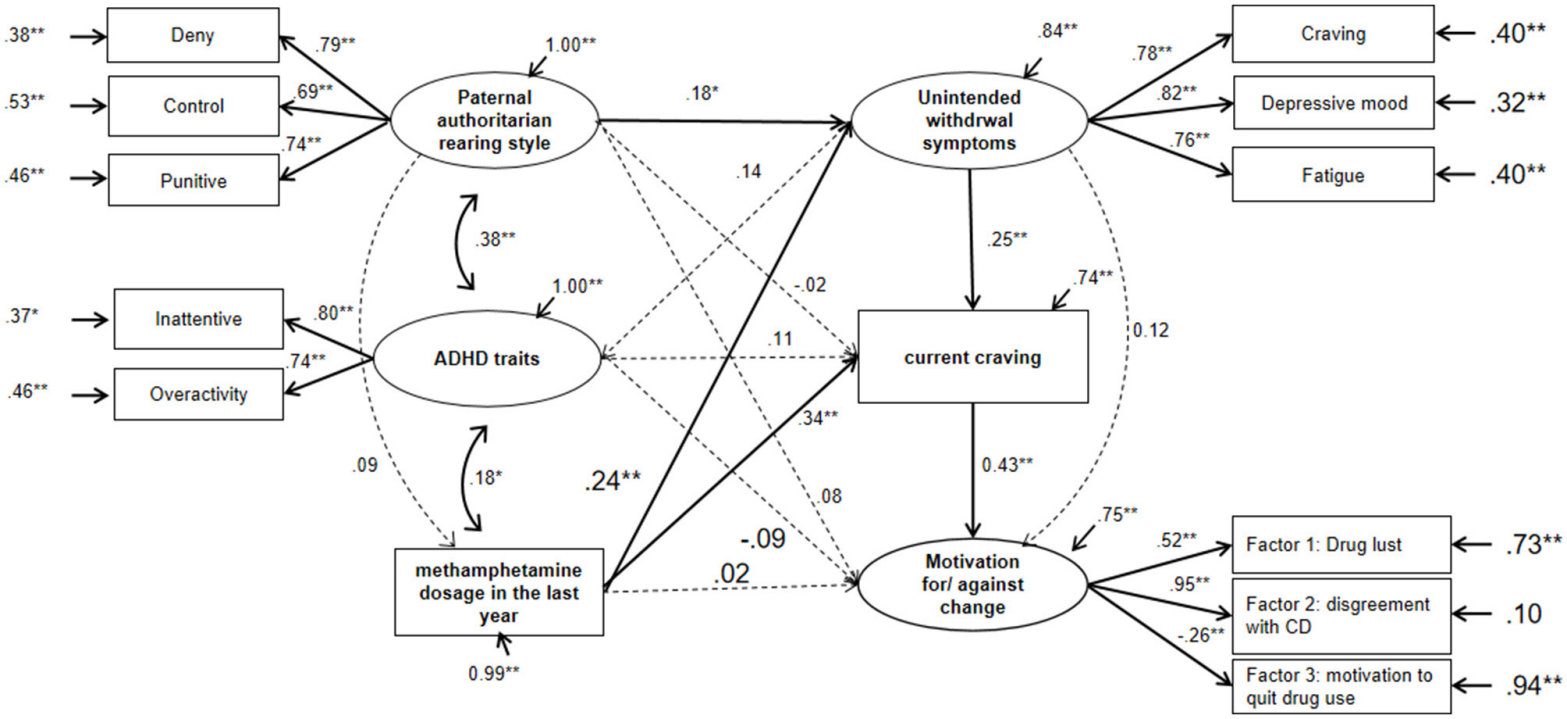
Calculated structural model of the influence factors for motivations for/against change. The statistics shown were the standardized parameters. For the purpose of clarity, the coefficients with a *P*-value of below 0.05 were displayed in solid lines, while those not reaching statistical significance were displayed in dotted lines. **P < 0.01, *P < 0.05, for the linear coefficients and errors.

The calculated structural equation model 1 is shown in Figure 4 and Supplementary Table 2. The parameters of the model (Supplementary Table 3) suggested good fitting. The solid lines represented the factor loading with a *p*-value of below 0.05, while the dotted lines represent the relationships that had not reached statistical significance. In the model, the paternal authoritarian rearing style interacted with unintended withdrawal symptoms and interacted affected the attitude to relapse, while ADHD traits were closely related both to the rearing style and to the last year of methamphetamine use dosage. The methamphetamine dosage, in turn, affected the motivation for/against change directly and indirectly *via* unintended withdrawal symptoms and current craving levels. Other relative models were examined (see Supplementary Tables 4–8) and showed acceptable fitting. In models 2–6 (Supplementary Tables 4–8 and Supplementary Figure 1), we mainly examined the relationship between maternal authoritarian rearing style, parental emotional warmth, ADHD traits, and impulsivity as measured by BIS-11. The results suggested that, like the father, an authoritarian mother also affected the withdrawal symptoms, while parental emotional warmth negatively correlated with BIS-11 impulsivity and current craving.

## 4. Discussion

Our research constructed a questionnaire that features both the motivation for and against change. Although most participants scored high in motivation for change and low in motivation against change, it is the motivation against change accounted for the largest loading in the model, which supported our first hypothesis and was in line with the other reports examining the relationship between change/counter-change talks and intervention outcomes (12, 13).

The structural equation models suggested that current MA craving may be the only direct influencing factor for motivation, explaining 18.5% variances. This result echoed the previous study carried out in voluntary detoxification patients, which suggested craving be the significant predictor for relapse (30). However, the self-reported craving level was relatively low. A previous study also reported low and stable levels of basal craving in a group of participants in a detoxification center (4). This might be explained by the contextual environmental effect on the craving, as in the isolated environment, the participants intuitive wanting for the drug has been successfully suppressed with the fact of drug unavailability. However, the actual desire for drugs might be higher as it was reflected in the motivation sub-scale. A study by Fan et al. (31) showed that the craving level could be elevated by providing drug-using videos, yet the elevated craving level is correlated with baseline craving (31), and BIS-11 measured impulsivity (32). In our study, we also found a relationship between BIS-11 motor impulsivity and non-planning with craving and a negative correlation between impulsivity (non-planning, cognition, and motor) with MFACD factor 3 (motivation to quit drug use), and non-planning with MFACD factor 2 (disagreement with CD).

Our study also showed that drug use history would interact with craving level independent of the CD duration. The last year's dosage of use not only related to the unintended withdrawal symptoms but also had a direct effect on the current craving. It should be noted that this measure not only reflected the biological intoxicating effect of the drug; more importantly, it was also a message of the addiction severity since higher dosages largely reflect more frequent use of the drug.

In the presented models, the parental authoritarian rearing style had an effect on the unintended withdrawal symptoms in our male participants. It suggested that negative parenting could have impacted emotional self-regulation, resulting in more significant experience of withdrawal symptoms. A number of surveys have linked childhood trauma with the development and exacerbation of substance use disorders (SUDs) in the form of deteriorated addictive symptoms, cravings, and relapse in both heavy drinkers (19, 33) and cocaine users (34, 35). Experiences with psychological, physical, or sexual abuse increase the risk of depressive disorders (36), antisocial personality disorders, and suicide attempts (37, 38). These psychiatric issues precipitate individuals to use addictive substances as an escape from current issues and increase the difficulty of SUD treatment. Subtler forms of psychological abuse, such as emotional neglect, may play a pivotal role in SUD relapse, as it was the only significant independent predictor of the first onset and recurrence of any depressive or comorbid disorder (36). The feature of emotional neglect has been incorporated into the authoritarian rearing style sub-scale of denial. Meanwhile, it also showed that parental emotional warmth might be protective by reducing current cravings. Parental emotional resources should not be overlooked even during the CD process.

Complicated premorbid and postmorbid factors contribute to the high relapse rates of SUDs. For example, individuals with attention-deficit/hyperactivity disorder (ADHD) seemed more likely to develop various addictive behaviors (17, 39–42). In patients with methamphetamine use disorders (MUDs), lifetime ADHD diagnoses were associated with obstacles to recovery, such as greater declines in instrumental activities of daily living, cognitive abilities, and social function (18), as well as more frequent psychosis episodes (43). Therefore, we assumed that ADHD traits might directly affect the level of craving and motivation. This was not confirmed in this study; instead, ADHD traits were found to be related to both paternal authoritarian rearing style and the last year's dosage of MA use. Therefore, the effect of ADHD traits on craving and drug-related motivation seemed to be indirect.

In this study, no participants reported symptoms that exceeded the suspected diagnostic line of ADHD, which contradicts reports of high comorbidity rates of ADHD and SUD (17, 44, 45). Furthermore, even though the scores on the symptom checklist were low in these cohorts, the relationship between psychiatric symptoms and unintended withdrawal symptoms, as well as craving levels, remained significant. Meanwhile, the self-report craving level was extremely low in the majority. Studies carried out in both compulsory setting and voluntary settings implicated that participants in the CD setting tended to downplay the severity of their problems, whereas voluntary patients, who should have had stronger motivation for change, reported a higher level of craving both at baseline and after visual drug context (46). Therefore, the estimation of psychiatric comorbidity rates in the compulsory setting should be made with caution, especially for conditions that might bring feelings of inferiority in the patients, such as ADHD, psychosis, and trauma histories.

Another limitation of the study was that the outcome of the participants was not traced, so it remained unknown for the efficacy of MFACD in predicting relapse. The participants' contact information was not required while the study was conducted. Therefore, the authors were not able to collect the follow-up information for these participants, which would be critical in following studies in examining the validity of the MFACD.

In conclusion, this study provided a measurement of motivation for and against change in subjects with drug misconduct and suggested that terms of motivation against change may disclose more psychological barriers than terms of motivation for change. Meanwhile, the model provided a broader view of the environmental–individual interaction of motivation and highlighted the importance of understanding individual brought-up history and drug use history. Together, the model with the measurement of motivation against change could be used for social workers who try to establish a closer relationship with the participants.

## Data availability statement

The raw data supporting the conclusions of this article will be made available by the authors, without undue reservation.

## Ethics statement

The studies involving human participants were reviewed and approved by the Ethics Committee of Ningbo Addiction Research and Treatment Center. The patients/participants provided their written informed consent to participate in this study.

## Author contributions

WS conceptualized the study, carried out the statistical analysis, and wrote the original draft. LL supported the study conceptualization, carried out interviews with the participants, and supported draft writing. YueL carried out interviews with the participants, supported the statistical analysis, and reviewed the draft. XX carried out interviews with the participants, supported them with resources, and reviewed the draft. WC supported with resources, assisted in project supervision, and reviewed the draft. HL supported with funding and project administration and reviewed the draft. WWZ supported in conceptualizing the study and funding acquisition, helped in method establishment and project administration, and reviewed the draft. YuL supported in study conceptualization, project administration, and draft review. HY supported in funding acquisition, supported in methodology establishment, program supervision, and draft review. WHZ led in funding acquisition and project supervision and supported in project administration, resource provision, draft writing, and discussion. All authors contributed to the article and approved the submitted version.
