# Supplementary figures and images for: The motivation against change in male methamphetamine users in the compulsory detoxification setting
Source: Front Psychiatry. 2023 Feb 6;14:1022926. doi: 10.3389/fpsyt.2023.1022926 (PMC9939637; doi:10.3389/fpsyt.2023.1022926)

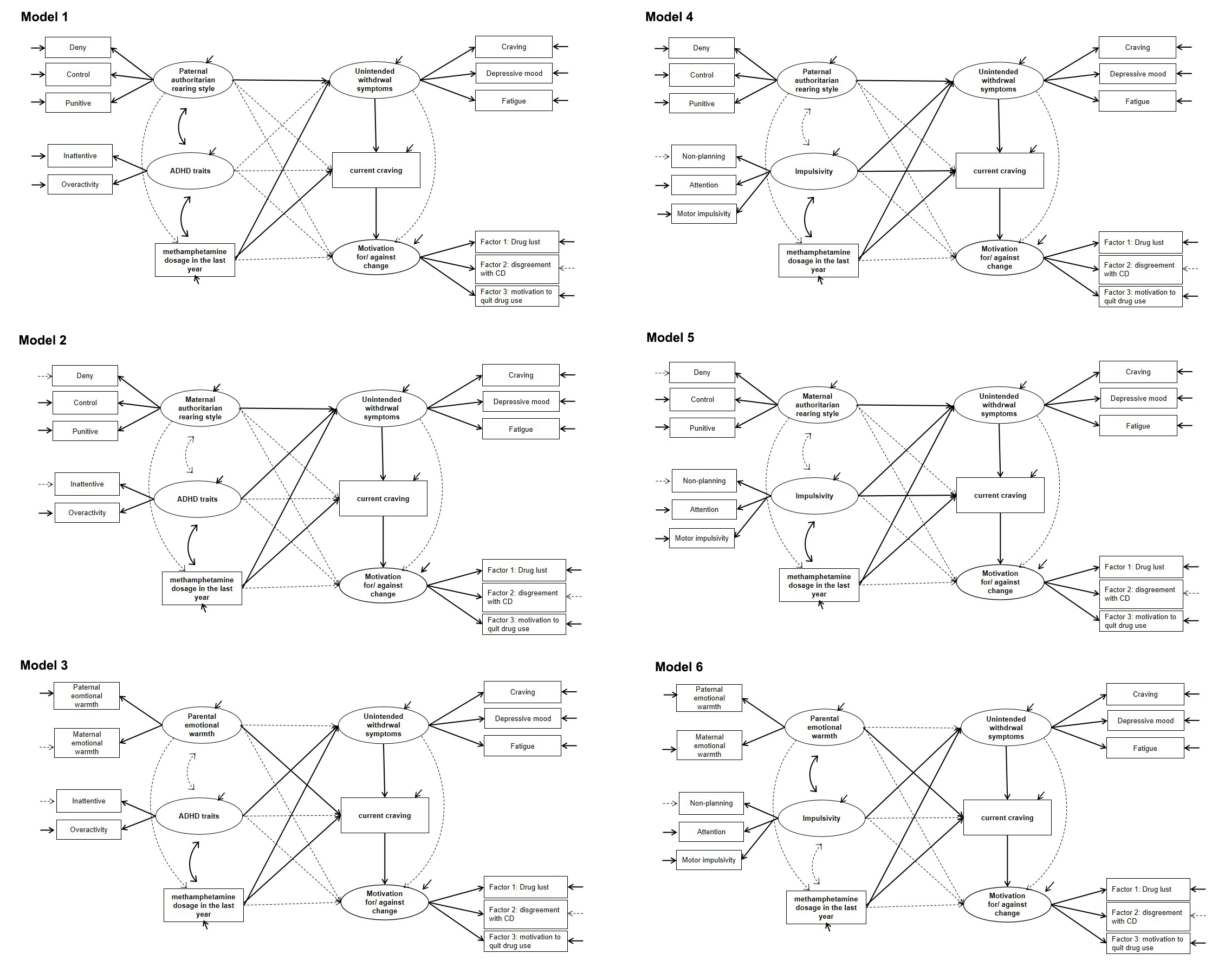

Supplement: Supplementary file 9 [file Image_1.TIF]
